# The Deadly Masquerade: Unveiling the Fatal Facade of the Great Imitator

**DOI:** 10.7759/cureus.86819

**Published:** 2025-06-26

**Authors:** Tyler R Ellett, Andrew Pippas, Humberto Rios, Seema B Jabbar

**Affiliations:** 1 Internal Medicine, St. Francis Hospital, Columbus, USA; 2 Hematology and Oncology, Piedmont Healthcare, Columbus, USA; 3 Gastroenterology and Hepatology, St. Francis Emory Healthcare, Columbus, USA; 4 Pathology, Piedmont Healthcare, Columbus, USA

**Keywords:** cholangiocarcinoma, igg4 disease, igg4-related cholangitis, primary sclerosing cholangitis (psc), sclerosing cholangitis

## Abstract

Sclerosing cholangitis (SC) is a fibroinflammatory condition that results in the progressive narrowing and destruction of bile ducts. It is generally classified into three distinct types: primary sclerosing cholangitis (PSC), secondary cholangitis, and immunoglobulin G4 (IgG4)-related cholangitis (IRC). IgG4-sclerosing cholangitis (IgG4-SC) is a rare entity characterized by inflammation and strictures within the biliary tree, arising from host-mediated immune responses. This condition represents the biliary manifestation of IgG4-related disease and may mimic other forms of sclerosing cholangiopathy, as well as cholangiocarcinoma. The clinical presentations of these diseases exhibit significant overlap, and diagnosis can be challenging. This case report discusses a 67-year-old male patient diagnosed with isolated IgG4-SC, who was initially suspected of having either cholangiocarcinoma or PSC.

## Introduction

Immunoglobulin G4 (IgG4)-related sclerosing cholangitis (IgG4-SC) is an autoimmune disease characterized by fibroinflammatory strictures of the hepatobiliary tract caused by IgG4. Due to the broad classification of IgG4-related disease (IgG4-RD), epidemiological data on IgG4-SC are limited and often indirect [1]. Most cases of IgG4-SC have been described as occurring alongside autoimmune pancreatitis (AIP), and genuinely isolated sclerosing cholangitis is estimated at around 0.28-1.08 per 100,000 [2]. IgG4-SC without AIP is often asymptomatic, with the most common presenting manifestation being jaundice [3,4]. Histopathological features of IgG4-RD include infiltration of IgG4+ plasma cells, storiform fibrosis, and obliterative phlebitis [5]. Available endoscopic techniques are key in differentiating stricture-related entities, as findings of symmetrical wall thickening in both the affected and unaffected biliary tract, along with the length of strictures, aid in distinguishing IgG4-SC from primary sclerosing cholangitis (PSC) and cholangiocarcinoma [6]. Isolated cases of IgG4-SC are frequently underreported in clinical literature, highlighting the need for heightened awareness and accurate diagnostic criteria. Timely and precise diagnosis is essential, as it can significantly influence treatment strategies and improve overall patient outcomes, emphasizing the importance of recognizing the unique features of this condition.

## Case presentation

**Figure 1:**
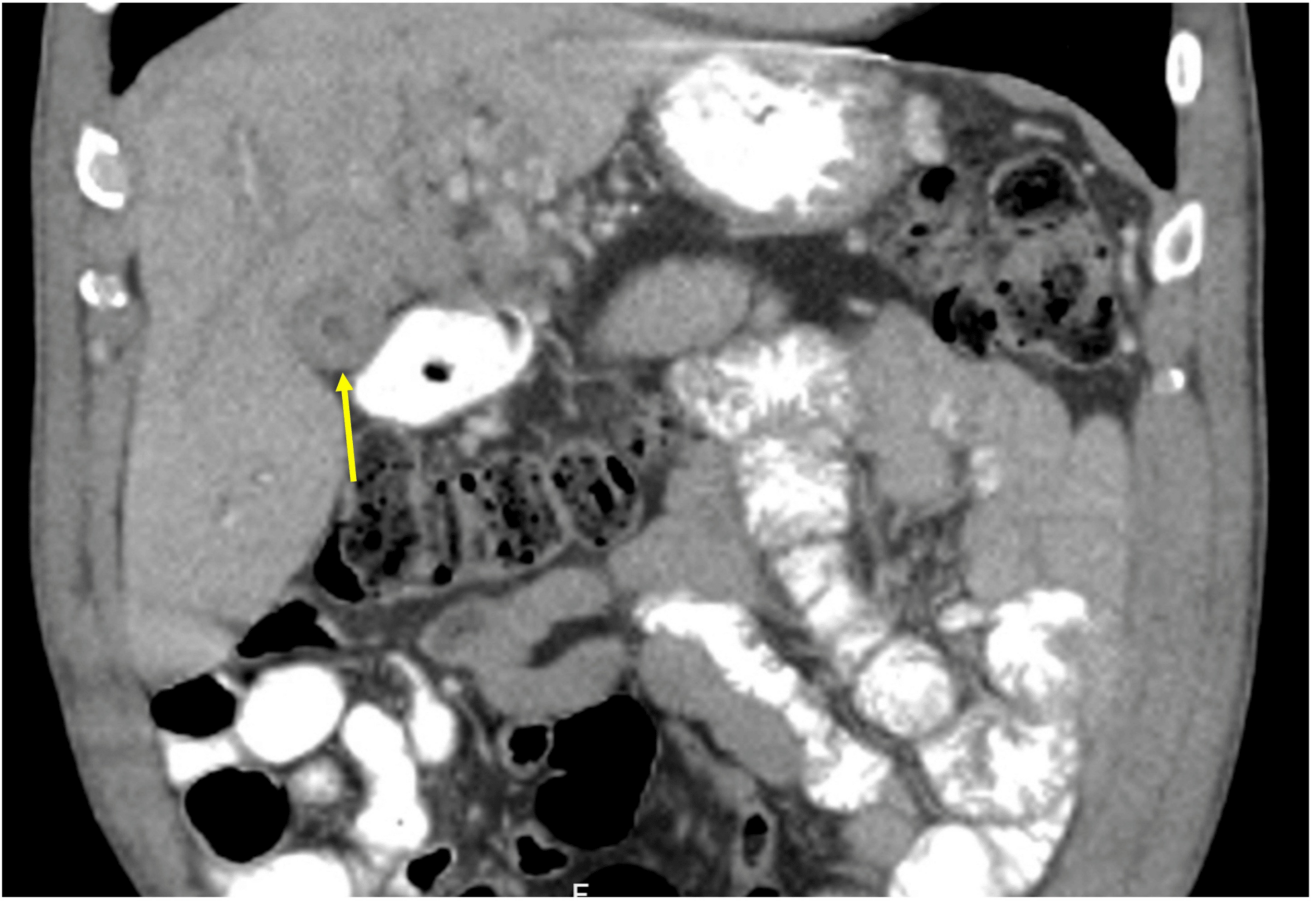
Computed tomography of nonspecific gallbladder wall thickening (yellow arrow).

**Figure 2:**
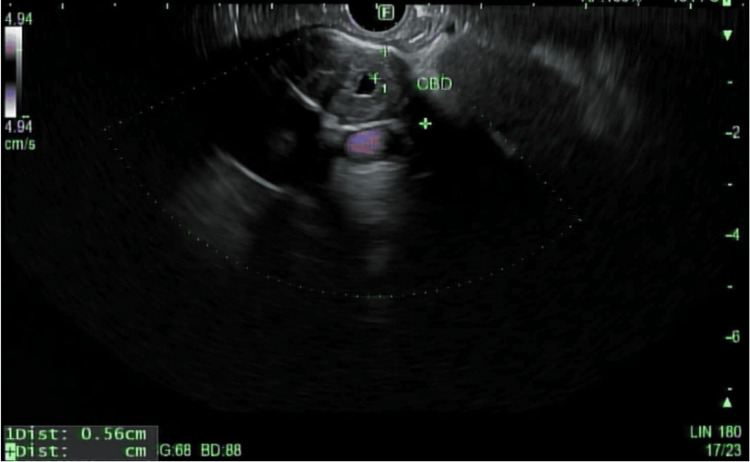
Endoscopic ultrasound demonstrating diffuse thickening of the common bile duct (CBD).

**Figure 3:**
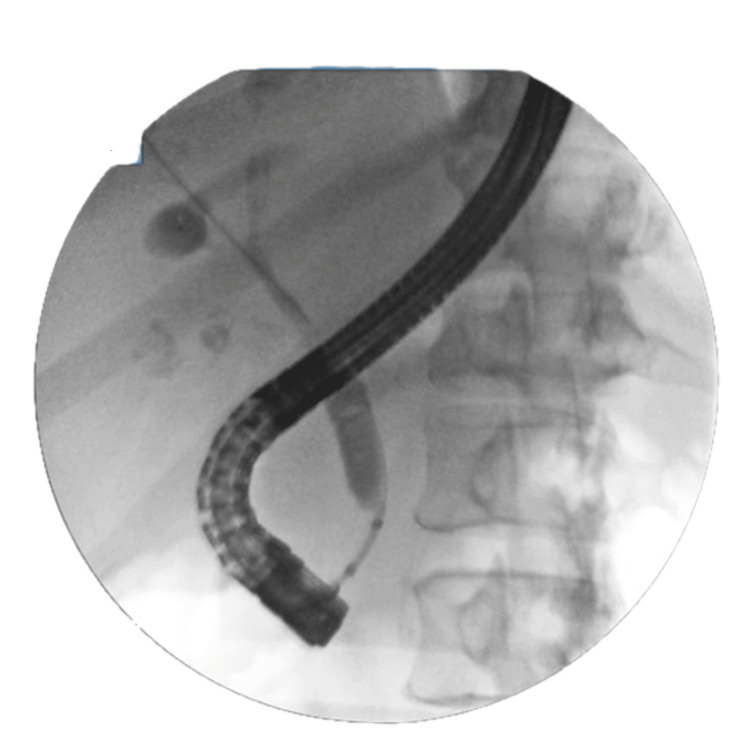
Bismuth type 1 stenosis (15 mm) of the common bile duct distal to the confluences of the right and left hepatic ducts.

**Figure 4:**
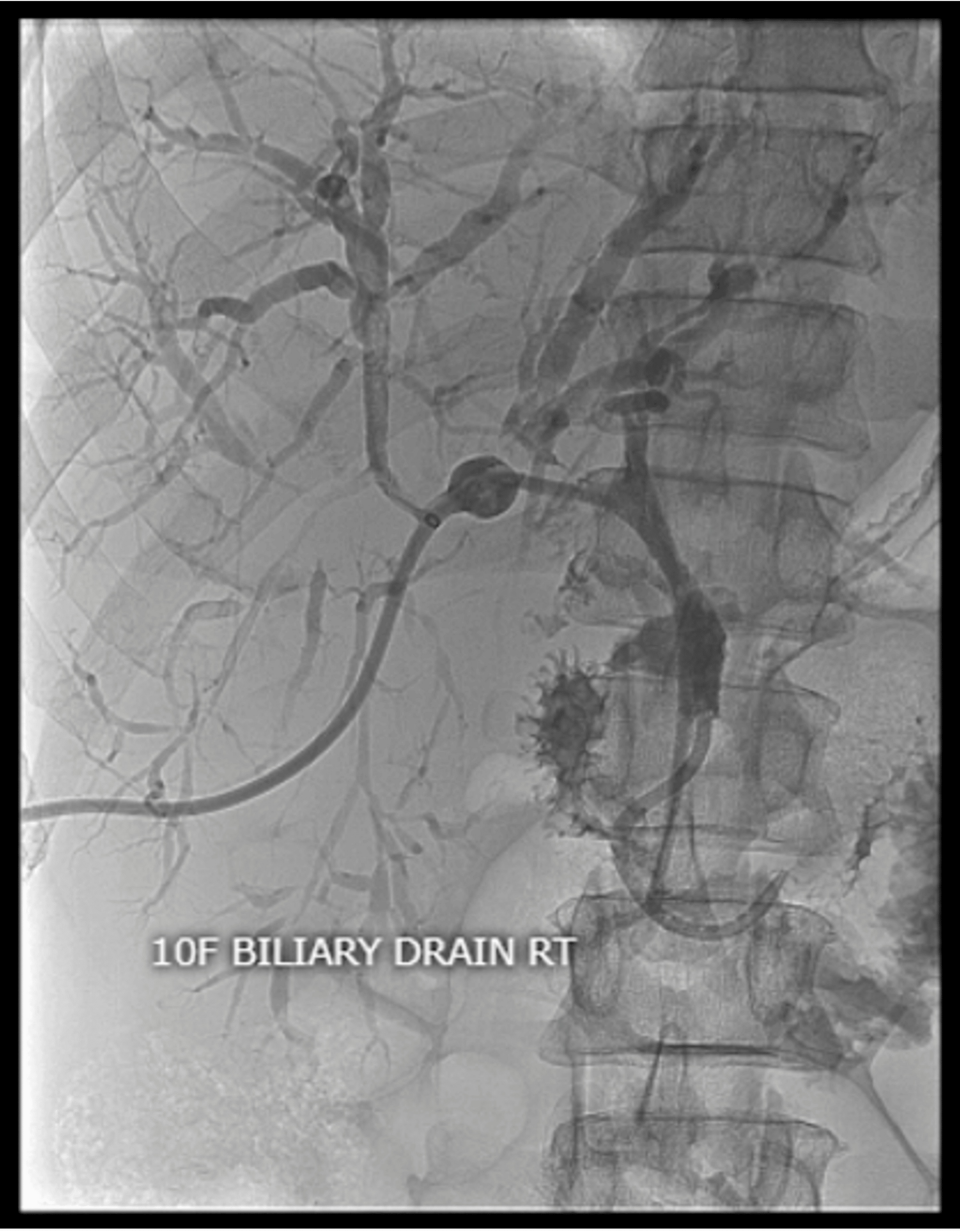
Percutaneous transhepatic cholangiogram demonstrating severe dilation of the intrahepatic biliary duct of the left lobe.

A 67-year-old African American male with a known past medical history of hypertension, diabetes mellitus, and anemia presented to the hematology and oncology service for evaluation of malignancy involving the gallbladder and left proximal hepatic duct. The patient’s initial presentation began with unintentional weight loss, jaundice, and nonspecific gallbladder thickening on computed tomography (Figure 1). Diffuse thickening of the common bile duct (CBD) and left proximal hepatic duct was observed, raising significant concern for periductal involvement. He was referred to gastroenterology for an endoscopic ultrasound (EUS), demonstrating two hyperechoic lesions in the left lobe of the liver. Celiac nodes were sampled at this time. Endoscopic retrograde cholangiopancreatography (ERCP) was also performed for strictures visualized by EUS involving the common hepatic duct (Figure 2). Throughout his clinical course, a total of five ERCP procedures were completed for stricture-related sequelae, such as jaundice, elevation of aminotransferases, hyperbilirubinemia, and sepsis. Several intrahepatic and extrahepatic ducts were involved, including the common bile duct, the common hepatic duct, both main hepatic ducts, and intrahepatic branches. Endoscopic treatment involved biliary sphincterotomy, balloon dilation, and stenting. A total of six stents were placed during this time. A Bismuth type 1 stricture measuring 15 mm was discovered in the common hepatic duct. ERCP and percutaneous transhepatic cholangiogram are shown in Figures 3, 4. Clotting and sludge were present in each case. No pancreatic ductal dilation was observed using various imaging techniques. Duodenal papilla biopsy was unrevealing. Carbohydrate antigen 19-9 (CA19-9) and carcinoembryonic antigen (CEA) levels were negative before surgical intervention.

All cytology brush sampling, fine needle aspiration of celiac lymph nodes, and biopsies of strictures were negative for malignancy. A colonoscopy was performed to evaluate microcytic anemia; high-grade dysplastic polyps were identified at the ileocecal valve. Lesions were deemed unamenable to endoscopic resection, and a right hemicolectomy and open cholecystectomy were completed. A wedge biopsy of the liver was also obtained, with findings significant for extensive acute cholangitis with sclerosing fibrosis associated with small to medium-sized bile ducts with obliteration and atrophy. No malignancy was seen. Severe acute and chronic fibrosing cholecystitis of the gallbladder was identified. The adenomatous polyp of the ileocecal valve was excised with clear margins. Twenty-five lymph nodes were resected without any evidence of metastatic carcinoma. During this time, a diagnosis of PSC was established. An interval MRI was obtained three months later with new findings of an infiltrating mass involving the porta hepatis, with probable metastatic lesions involving the left dome. There was concern for cholangiocarcinoma involving the common hepatic duct, and an ultrasound-guided liver biopsy revealed hepatic parenchyma with lymphoplasmacytic inflammation and fibrosis with a storiform pattern. No definite bile duct injury was identified in the sampled material. Differentials included IgG4-related sclerosing disease, primary sclerosing, autoimmune process, and inflammatory pseudotumor.

**Figure 5:**
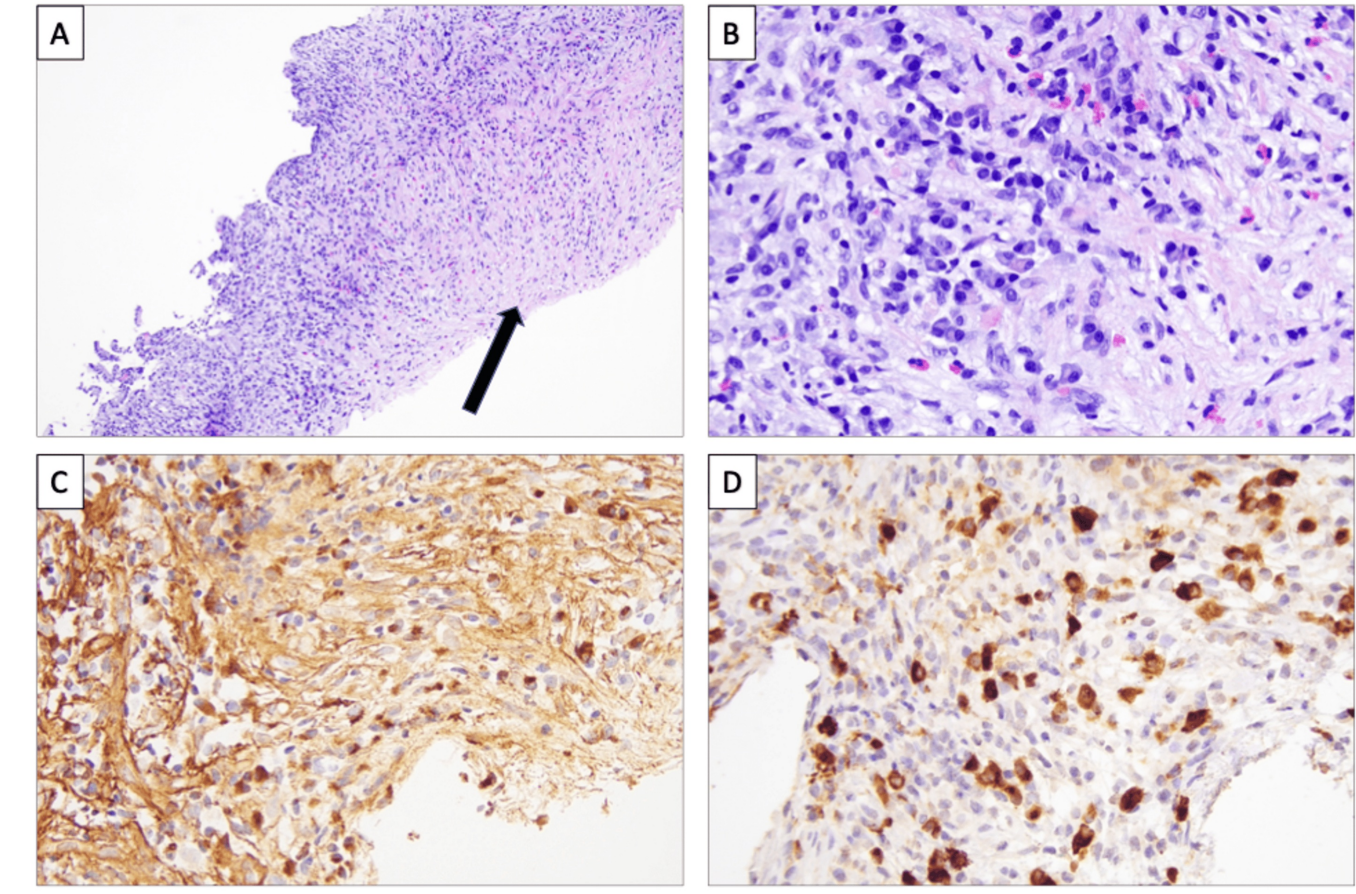
Hepatic parenchyma with lymphoplasmacytic inflammation and fibrosis. A. Histologic sections show an area of fibrosis (arrow) adjacent to areas of chronic inflammation (10x, H&E). B. Chronic inflammation shows an increased number of plasma cells admixed with few eosinophils and lymphocytes (40x, H&E). C. IgG4 immunohistochemical stain shows high background staining and underestimates the number of plasma cells (40x, IgG IHC). D. IgG4 immunohistochemical stain highlights IgG4+ plasma cells, which outnumber plasma cells for IgG. The number of IgG4 plasma cells in this one high-power field is 44 (40x, IgG4 IHC). H&E: hematoxylin & eosin; IgG4: immunoglobulin G4; IHC: immunohistochemistry.

Histopathologic identification of focally greater than 40 IgG4-positive plasma cells per 40x high-power field was seen. IgG4/IgG plasma cell ratio was limited due to the IgG immunohistological stain, which showed high background staining and an underestimated number of plasma cells present, as IgG4+ plasma cells outnumbered plasma cells staining for IgG (Figure 5). The staining intensity of atypical perinuclear antineutrophil cytoplasmic antibodies ranges from 30% to 80%. Human leukocyte antigen DRw52a was positively detected. IgG4 disease was diagnosed in this patient, and high-dose steroids were initiated. Since a high-dose steroid taper was completed, total IgG levels, bilirubin, and aminotransferase levels have returned to normal limits. The IgG4 immunoglobulin subclass remains elevated but is trending down. He is on maintenance therapy with Cellcept 1000 mg twice daily.

## Discussion

Sclerosing cholangitis (SC), defined as the progressive narrowing and destruction of bile ducts, was previously categorized into PSC and secondary SC. In the last decade, IgG4-SC has been established as a distinct clinical phenotype of SC. IgG4-SC is generally a male-dominant disease, with an average age of presentation in the sixth decade of life [5,7]. There are currently two widely accepted revised diagnostic criteria: the HISORt criteria and those established by the Japanese Biliary Association. Both share the same five elements yet differ in how they define certain and probable diagnoses based on the combination of a typical cholangiogram, laboratory findings of elevated IgG4 antibodies, presence of systemic involvement, histological examination, and response to steroids. The coexistence of AIP and classification of IgG4-SC based on cholangiography must be established first, as both have separate diagnostic criteria and differential diagnoses. Given the strong association with AIP, its presence supports IgG4-SC over malignancy. IgG4-SC is subclassified into four distinct types based on the location of biliary strictures: type 1: strictures in the distal bile duct; type 2a: intrahepatic and extrahepatic strictures with pre-stenotic dilations; type 2b: intrahepatic and extrahepatic strictures without pre-stenotic dilations; type 3: hilar strictures combined with distal choledochal involvement; and type 4: isolated hilar strictures [8].

Cholangiocarcinoma (CC) is the principal differential diagnosis of all types of IgG4-SC, except type 2. Chronic pancreatitis and pancreatic cancer should be differentiated from type 1 and PSC from type 2. Isolated IgG4-sclerosing cholangitis is uncommon, representing roughly 4% of all IgG4-SC cases. Of these, 30-50% of isolated IgG4-SC instances show a type 4 classification on cholangiography [9]. In our case, isolated IgG4-SC type 1 is initially identified with interval strictures typical of type 2a. IgG4-related cholangitis (IRC) is often under-recognized and misdiagnosed, as no single diagnostic test distinguishes it from PSC or CC. Misdiagnosing IRC can lead to inappropriate medical treatments and potentially harmful surgical interventions.

Modalities such as IgG4 levels, CT, MRI, EUS, and intraductal ultrasonography (IDUS) are highly effective in differentiating between IgG4-SC, PSC, and cholangiocarcinoma. Nearly all patients with IgG4-SC exhibit elevated IgG4 levels; values four times the upper normal limit are 100% specific for IgG4-SC [6]. For example, in our patient, the IgG4 level was measured at 817. However, some patients with isolated subtypes may not display significant increases in IgG4 levels compared to those associated with AIP, rendering IgG4 levels insufficient for diagnosis.

Noninvasive imaging techniques, such as CT and magnetic resonance cholangiopancreatography (MRCP), aid in identifying specific findings, including bile duct wall thickening. In cases of isolated IgG4-SC, Yata et al. reported that CT findings of circumferential single-layer contrast-enhanced symmetric wall thickening in the bile ducts show 80% sensitivity and specificity for differentiating isolated IgG4-SC from CC. In contrast, dual-layered contrast enhancement in the bile duct wall is 90% specific for CC [10].

CT and MRI also noted many incidental findings. A solid mass of the right posterior pole kidney, suggestive of renal cell carcinoma, was seen and ablated. It was suspected that his findings of multiple bilateral lung pulmonary nodules represented metastatic foci. Nonspecific prominence of a left groin lymph node and thickening of the gallbladder wall were also seen. In retrospect, these findings were likely related to the patient’s IgG4-SC as lymphadenopathy, solid lesions of the kidneys, and lung nodules are commonly seen in IgG4-RD [11]. Approximately 51% of patients with IgG4-SC show gallbladder involvement, typically presenting as diffuse wall thickening and enhancement [12]. ERCP is still the gold standard for differentiating bile duct stenosis. Typical findings of IgG4-SC on a cholangiogram include segmental strictures (>3 mm) and long strictures (>10 mm) with pre-stenotic dilation, as well as strictures of the distal common bile duct [1]. In IgG4-SC, the thickening of the bile duct wall is usually circular, homogeneous, and smooth, extending beyond the area of stenosis. It is better visualized using IDUS than EUS [13]. There is variability in non-stricture regions among patients with or without autoimmune pancreatitis. A few cases of isolated subtypes have been reported without thickening in unaffected areas [14].

Unlike PSC and CC, IgG4-SC is very responsive to steroid therapy. Guidelines recommend oral steroid therapy with 0.6 mg/kg/day, followed by maintenance dosing of 2.5-5 mg/day for six to 36 months [15]. Despite good outcomes with the use of steroids, long-term outcomes remain uncertain. Patients are prone to disease progression, relapse, and cancer during maintenance therapy. Approximately 31-57% will experience relapse during steroid tapering. In addition, patients with more proximal common bile duct strictures are associated with higher recurrence rates (i.e., types 2-4 IgG4-SC) [16]. Recently, a large multicenter study conducted by Kurita et al. recognized that patients with AIP and IgG-SC are associated with a high risk of developing pancreatic and bile duct cancer [17]. There is currently no research on isolated IgG4-SC and its risk of pancreaticobiliary cancer, likely due to the limited studies on this topic. A novel CD-19-targeted therapy has been shown in 2024 to reduce the risk of IgG4-related disease flares. These focused approaches may one day replace the need for long-term glucocorticoid immunosuppression [18].

## Conclusions

IgG4-SC is an autoimmune disorder classified by biliary tract stricture location and the presence or absence of autoimmune pancreatitis. Given the broad classification of IgG-related disease, epidemiological data on isolated IgG4-SC are underreported. Widely accepted diagnostic criteria, such as the HISORt criteria and those established by the Japanese Biliary Association, are used in diagnosis. IgG4 levels alone are insufficient to rule out the diagnosis of IgG4-RD, especially in isolated cases. ERCP is considered the gold standard for differentiating bile duct stenosis, while IDUS is superior for assessing bile duct characteristics. Wall thickening past the stricture location is considered pathognomonic, but not always seen in isolated cases of IgG4-SC. Computed tomography is a reliable noninvasive modality. Circumferential single-layer contrast-enhanced symmetric wall thickening is moderately specific for isolated IgG4-SC. Glucocorticoids are crucial in treating these patients and can help differentiate IgG4-SC from PSC. Biologics are showing promising results and may offer new steroid-sparing options for the treatment of IgG4-RD.
